# Is the maternal risk of pregnancy acceptable in patients with moderate pulmonary hypertension?

**DOI:** 10.1177/20458940211023697

**Published:** 2021-06-15

**Authors:** Farid Rashidi, Seyed Ali Mousavi-Aghdas, Cihangir Kaymaz

**Affiliations:** 1 Tuberculosis and Lung Disease Research Center, 48432 Tabriz University of Medical Sciences, Tabriz, Iran; 2 Department of Cardiology, Kosuyolu Heart Education and Research Hospital, University of Health Sciences, Istanbul, Turkey

Dear editor,

We read with great interest a recent study by Weisi Lai et al., published in the journal of *Pulmonary Circulation*.^1^ The authors concluded that pregnancy carried favorable outcomes in women with moderate pulmonary hypertension (PH), diagnosed by transthoracic echocardiography (TTE).^1^ However, respectfully, we believe this conclusion might be misleading because of multiple reasons:

- Ignoring the underlying condition(s) leading to PH
- Using only TTE for diagnosis and staging of PH in pregnant women
- A wide patient inclusion time limit (2004–2016)
- Administering medications with potentially hazardous effects on mother or fetus during pregnancy

In this series, prevalence of idiopathic pulmonary arterial hypertension (PAH) was not addressed, while congenital heart disease and rheumatic heart disease were noted in 56.4% and 33.3.% of mild PH patients and in 75.5% and 20.4% of severe PH patients, respectively. The PAH-targeted therapies were documented in 6.1% of pregnant women with mild PH and in 41.1% of severe PH, respectively. More interestingly, not only tadalafil or sildenafil but also bosentan seemed to be continued through gestation. None of the patients with severe PH had functional class IV status before pregnancy, although class IV status was noted in 16.4% at admission, in 12.2% after delivery, and in 26.5% at the end of follow-up. At the final assessment, the frequency of class IV status among women with severe PH was 10 times higher than those with moderate PH. In this study, poor cardiac function before pregnancy, irregular antenatal care, and hyperuricemia were found to predict mortality in pregnant women with PH. Therefore, these results were concluded to suggest an acceptable risk of pregnancy in women with moderate PH as quantitated by echocardiography.

In the past, there was a considerable possibility of death for pregnant women with PH (25–56%).^2^ It is shown that pulmonary artery pressure rises through pregnancy and especially in patients with severe PH and puts the patient and fetus in jeopardy.^3^ On the contrary, it has been proposed that patients who have a well-controlled disease and are long-term responders to calcium channel blockers may go through pregnancy safely.^4^ Despite the advances in treatment, PH continues to be recognized as a contraindication to pregnancy by the 2015 European Society of Cardiology guidelines (Recommendation IC).^5^ However, with recent improvements in the outcomes of patients, concerns have been raised to reconsider this recommendation.

In a large cohort of 151 pregnant women with PH, 43% of the mortality was attributable to idiopathic PAH.^6^ Because of the heterogeneous outcomes, we strongly believe that PH patients should be approached individually according to their underlying condition(s).

A meta-analysis demonstrated a correlation coefficient of 0.70 between systolic pulmonary arterial pressure (PAP) measured by TTE and right heart catheterization (RHC). In this study, the sensitivity and specificity of TTE for diagnosis of PH were 83% and 72%, respectively.^7^ On the other hand, there is a poor agreement between echocardiographic mean PAP estimates (four tricuspid regurgitation jet-derived) and RHC measurement, regardless of the underlying pathology.^8^ Thus, in concordance with the latest guidelines from the European Society of Cardiology and European Respiratory Society, TTE should only be used as the first-line screening modality.^5^ With improvements in the echocardiographic evaluation of the right ventricular function, TTE has become a valuable tool in the follow-up of patients.^9^ However, RHC is still the gold standard test, and considering its safety, it should be performed for a definitive diagnosis of PH.^5^ Additionally, it was previously shown that in contrast to RHC, TTE overestimates the mean PAP significantly in pregnant women and 32% of patients diagnosed with PH are in fact normal but misclassified.^10^ Unfortunately, in the study by Weisi Lai et al., RHC has not been performed for patients.

During the past decade, effective medications have been developed or approved for the treatment of PH. These medications have affected the outcomes by reducing mortality, increasing exercise capacity, and improving hemodynamics.^5^ The study by Wiesi Lai et al. included patients from 2004 to 2016. Thus, in the sampling of patients, it should have been considered that more recent patients probably had better outcomes than did the older patients.^11^ Although long-term mortality was significantly higher in women with severe PH compared with moderate PH, this study tells us nothing regarding the frequency of increased mortality within the first days following delivery and management strategies.

In conclusion, although the mortality of PH has dropped significantly over the years, severe PH and idiopathic PAH continue to prohibit pregnancy. In summary, the management of PH should be individualized based on the underlying conditions(s), RHC findings, and response to treatment. This is especially true in pregnant patients. Large multi-center studies are needed to find the influence of recent advances in the management of PH in pregnant women with PH.

## References

[1] Lai W Ding Y Wen L. Long-term outcomes of pregnant women with pulmonary hypertension diagnosed by echocardiography: a retrospective cohort study in a single center from China. Pulmon Circ 2021; 11: 2045894020966876. 10.1177/2045894020966876 PMC7869154 33614014

[2] Rex S Devroe S. Anesthesia for pregnant women with pulmonary hypertension. Curr Opin Anaesthesiol 2016; 29: 273–281. 26978591 10.1097/ACO.0000000000000310

[3] Katsuragi S Yamanaka K Neki R , et al. Maternal outcome in pregnancy complicated with pulmonary arterial hypertension. Circ J 2012; 76: 2249–2254. 22785004 10.1253/circj.cj-12-0235

[4] Jaïs X Olsson KM Barbera JA , et al. Pregnancy outcomes in pulmonary arterial hypertension in the modern management era. Eur Resp J 2012; 40: 881–885. 10.1183/09031936.00141211 22282544

[5] Galiè N Humbert M Vachiery J-L , et al. 2015 ESC/ERS Guidelines for the diagnosis and treatment of pulmonary hypertension: the Joint Task Force for the Diagnosis and Treatment of Pulmonary Hypertension of the European Society of Cardiology (ESC) and the European Respiratory Society (ERS): Endorsed by: Association for European Paediatric and Congenital Cardiology (AEPC), International Society for Heart and Lung Transplantation (ISHLT). Eur Heart J 2015; 37: 67–119. 26320113 10.1093/eurheartj/ehv317

[6] Sliwa K van Hagen IM Budts W , et al. Pulmonary hypertension and pregnancy outcomes: data from the Registry of Pregnancy and Cardiac Disease (ROPAC) of the European Society of Cardiology. Eur J Heart Failure 2016; 18: 1119–1128. 10.1002/ejhf.594 27384461

[7] Janda S Shahidi N Gin K , et al. Diagnostic accuracy of echocardiography for pulmonary hypertension: a systematic review and meta-analysis. Heart 2011; 97: 612–622. 21357375 10.1136/hrt.2010.212084

[8] Kaymaz C Yasar Akbal O Hakgor A , et al. Reappraisal of the reliability of Doppler echocardiographic estimations for mean pulmonary artery pressure in patients with pulmonary hypertension: a study from a tertiary centre comparing four formulae. Pulmon Circ 2018; 8: 1–9. 10.1177/2045894018762270 PMC5865458 29480067

[9] Wright LM Dwyer N Celermajer D , et al. Follow-up of pulmonary hypertension with echocardiography. JACC Cardiovasc Imaging 2016; 9: 733–746. 27282440 10.1016/j.jcmg.2016.02.022

[10] Penning S Robinson KD Major CA , et al. A comparison of echocardiography and pulmonary artery catheterization for evaluation of pulmonary artery pressures in pregnant patients with suspected pulmonary hypertension. Am J Obstet Gynecol 2001; 184: 1568–1570. 11408882 10.1067/mob.2001.114857

[11] Pieper PG Lameijer H Hoendermis ES. Pregnancy and pulmonary hypertension. Best Pract Res Clin Obstet Gynaecol 2014; 28: 579–791. 24685319 10.1016/j.bpobgyn.2014.03.003

